# Comprehensive Analysis of Predictors and Outcomes in Breast Cancer Screening in Romania: Insights from Demographic, Clinical, and Lifestyle Factors

**DOI:** 10.3390/jcm14051415

**Published:** 2025-02-20

**Authors:** Oana Maria Burciu, Ioan Sas, Adrian-Grigore Merce, Simona Cerbu, Aurica Elisabeta Moatar, Adrian-Petru Merce, Ionut Marcel Cobec

**Affiliations:** 1 Doctoral School, Faculty of Medicine, “Victor Babes” University of Medicine and Pharmacy Timisoara, 300041 Timisoara, Romania; 2 Department of Functional Sciences, Medical Informatics and Biostatistics Discipline, “Victor Babes” University of Medicine and Pharmacy, 300041 Timisoara, Romania; 3 Department of Obstetrics and Gynecology, “Victor Babes” University of Medicine and Pharmacy, 300041 Timisoara, Romania; 4 Department of Cardiology, Institute of Cardiovascular Diseases, 300310 Timisoara, Romania; 5 Discipline of Radiology and Medical Imaging, “Victor Babes” University of Medicine and Pharmacy Timisoara, 300041 Timisoara, Romania; 6 ANAPATMOL Research Center, Faculty of Medicine, “Victor Babes” University of Medicine and Pharmacy Timisoara, 300041 Timisoara, Romania; 7 Clinic of Internal Medicine-Cardiology, Klinikum Freudenstadt, 72250 Freudenstadt, Germany; 8 Department of Cardiovascular Surgery, Institute of Cardiovascular Diseases, 300310 Timisoara, Romania; 9 Clinic of Obstetrics and Gynecology, Klinikum Freudenstadt, 72250 Freudenstadt, Germany

**Keywords:** breast cancer, screening, mammography, breast ultrasound, biopsy, regions

## Abstract

**Background/Objectives:** The primary purpose of this study is to provide a more in-depth insight into various demographic, clinical, and lifestyle factors in relation to breast cancer and to predict the extent to which certain variables described as “predictors” might lead to further investigation. By analyzing a large cohort, we are able to provide valuable and up-to-date information on breast cancer screening, support breast specialists, and further enhance international screening guidelines. **Methods:** We screened for breast cancer in a population of women aged 50 to 69 years by using the standardized breast cancer imaging screening method (breast mammography) and ultrasonography as a complementary imagistic tool, and we compared the results with the gold standard, breast biopsy. For this, 58,760 women with no known history of breast cancer coming from 4 major regions of Romania (North-East, North-West, South-East, and West) were first evaluated through mammography. Out of these, 3197 women with positive mammograms subsequently underwent a breast ultrasound examination. The remaining 688 patients with positive breast ultrasound were further referred for a breast biopsy. **Results:** The statistical analysis revealed several predictors such as the body mass index (BMI), positive family medical history of breast cancer, age at first birth, and age at menopause that influenced the progression from mammography (first stage of the screening program) towards echography (additional imaging modality). Furthermore, we established that age, age at first birth, and BMI are significant predictors of progression from echography towards biopsy (the last stage of the screening program). Furthermore, by analyzing the number of positive biopsies (688) out of the total number of patients in the study (58,760), we calculated a total breast cancer detection rate of 8 per 1000 patients. Lastly, by studying the patient demographics in the context of breast cancer (BC) screening, we observed that participants coming from an urban environment presented a higher rate of positive mammographic results as compared to ones of rural provenience. **Conclusions:** Our study analyzed a large cohort of patients and offers real world data which shows that multiple factors were positively associated with an increased risk of BC. Older age, older age at first birth, and an older menopausal age are all estrogen-dependent risk factors that were linked with an increased breast cancer risk in our study. Furthermore, our findings concerning the rural/urban disparities and regional differences highlight the need for region-specific interventions to address lifestyle risk factors, improve healthcare access, and enhance breast cancer screening and follow-up protocols, particularly in underserved areas like the North-East and South-East regions.

## 1. Introduction

Breast cancer (BC) is an undeniable public health concern affecting well-developed and less developed countries, exhibiting a strenuous mark on the economy, families, and healthcare providers all over the world. The recent statistics place BC as the most prevalent cancer affecting women and the second cause of death by cancer, after lung cancer. Therefore, BC screening is crucial for minimizing healthcare expenses and improving prognosis and overall survival in cancer patients by detecting the disease at an early stage, which is easier to treat. Studies in the literature have analyzed survival in both women and men (approximately 1% of breast cancers occur in men), the results suggesting better survival outcomes in women compared to men and an older age at diagnosis in men [1,2,3]. 

The objective of this study was to consolidate the existing research data on BC screening, ultimately aiming for improved disease management. This study makes a unique contribution by providing a large-scale analysis of BC screening data collected from diverse regions of Romania, a country with no previous nationally implemented screening program. By identifying demographic, reproductive, and lifestyle factors associated with screening progression, as well as regional disparities, this research offers insights that are directly applicable to Romania and may inform BC screening strategies in other countries with similar resource constraints or sociocultural profiles. Additionally, the study’s emphasis on predictors of progression through initial mammographic screening → additional breast ultrasound → biopsy highlights critical decision-making factors that can enhance clinical guidelines. Therefore, our approach addressed BC as an epidemiological challenge, considering various variables that may impact the development of the disease and comparing the most used standard screening methods. Various modifiable and non-modifiable factors have demonstrated different ways in which they can potentially increase the risk of BC. The link between hormonal exposure and BC has been thoroughly investigated, as approximately 70% of breast cancers are hormone-dependent tumors (expressing progesterone or/and estrogen receptors). When a tumor does not express hormone receptors, it is referred to as a triple-negative tumor. These tumors tend to have an overall poor prognosis due to being unresponsive to hormone therapies, such as CDK4/6 inhibitors, which have revolutionized cancer treatment. Triple-negative tumors are prone to rapid metastasis and are highly aggressive [4,5,6,7]. 

In various studies, age is considered to be the second most important risk factor for BC after gender, as BC incidence increases with age, peaking at menopausal age, and then gradually decreasing or remaining constant. This issue can also be attributed to the steadily increasing life expectancy in most developed countries, with the median age for developing BC now considered to be around 60 years, with slight variations. A later menopause onset has also emerged as a risk factor due to prolonged exposure to endogenous estrogen. When discussing BC outcomes in older patients, the results are conflicting, with some studies suggesting that older patients experience worse outcomes [8,9,10]. 

Early menarche onset is another factor correlated with a higher risk of developing BC due to earlier exposure to the hormonal changes that induce the beginning of menstrual cycles. In some studies, earlier menarche onset was also associated with more aggressive tumoral characteristics [4,11]. 

The body mass index (BMI) has been shown to influence, to a certain degree, the outcomes and prognosis of BC patients, as well as cancer development itself. Therefore, multiple studies addressed the issue of patients with a high BMI and its adverse effects on disease-free survival and overall survival. Some research papers indicated interesting observations concerning more aggressive tumoral BC characteristics in obese patients or associations between a higher BMI and the risk of developing BC in postmenopausal women. Other studies indicated a possible link between obesity and BC recurrence, perhaps due to increased survival, the persistence of residual tumor cells, and the ability of adipose cells to synthesize estrogen [12,13,14,15]. 

A positive family medical history (FMH) of BC is a crucial aspect to be taken into consideration when interviewing a patient during a breast consultation. Nearly all research data to date agree that a positive history of BC can increase a patient’s chances of developing the disease, with some exceptions—for example, a mother developing BC after the age of 50 does not particularly increase the risk of developing cancer in a daughter. In women with positive BC FMH or multiple cases of BC among first-degree relatives, more in-depth investigations need to be performed, including testing for the most common mutations associated with breast cancer and implementing an earlier and more intensive screening process. One study emphasized that a BC FMH of breast cancer mainly influenced the patient’s age, tumor stage, and grade at diagnosis [16]. 

Older age at first childbirth is an established risk factor for BC, with conflicting results regarding the prognosis of BC. However, this well-established risk factor raises questions about the overlapping increasing maternal age at first birth and the growing number of BC cases [17,18]. 

Several original studies and literature reviews emphasize the protective effects of breastfeeding against BC. One study notes that for every 12 months of breastfeeding, the risk of BC decreases by 4.3%, which is added to the 7.0% decrease in risk observed for each birth. Breastfeeding has been shown to reduce the risk of triple-negative breast cancer (20%) and improve cancer outcomes in patients with BRCA mutations (22–50%) [19,20,21]. 

Hormonal treatment in menopausal women has been associated with an elevated risk of BC compared to those who have never used hormone therapy. In one study, the risk increase persisted for more than a decade after the menopausal hormonal treatment was ceased and the risk was greater for estrogen–progestogen combinations than for estrogen-only preparations (especially with daily use of progestogen) [22,23]. 

Another study assessed the use of hormonal therapy in hysterectomized women. The results showed that hysterectomized women should be prescribed estrogen-only therapy, while women who did not undergo surgery should use combined estrogen–progestogen therapy with progestogens that have the lowest risk [24]. 

Smoking and alcohol use have also been studied in relation to the possibly increased risk of developing breast cancer. Follow-up studies and literature reviews reported that cigarette smoke exposure led to an increased risk of several types of BC [25]. One study showed an increase in BC risk in BRCA1 and BRCA2 carriers that smoked for more than 5 years during their pre-reproductive years [26]. 

As previously mentioned, multiple modifiable and non-modifiable factors have to be taken into consideration when properly interviewing participants in a BC screening program. After a well-conducted anamnesis of the patient, two imagistic screening tools, mammography and breast ultrasound, are used, either separately or combined depending on the case. Current guidelines, however, agree that mammography remains the first choice in terms of BC screening for average-risk women, breast ultrasound being regarded as a complementary imaging tool. In one study, mammography had an overall high sensitivity of 85%, which was negatively affected by dense breast tissue. In the same study, breast ultrasound presented higher sensitivity than mammography, and the combination of mammography and ultrasound showed superior sensitivity compared to either method alone [27,28,29]. 

When discussing BC screening guidelines, there are slight conflicting views on when a woman should begin screening, depending on her personal medical history, age, and various other factors. Most screening programs usually recommend a biannual mammographic screening of women aged 50 to 69. Earlier or annual mammographic screening started at or below 40 years old can be inefficient for average-risk women with dense breast tissue. However, the screening should be annual and start at 40 years old or even earlier in certain cases—a positive family history of breast cancer or genetic mutations can lead to a higher BC risk. The most common and well-known mutations that increase the risk of BC—by up to 85%—are BRCA1 and BRCA2 mutations, which are also associated with a 45% risk of developing ovarian cancer during a woman’s lifetime. Nevertheless, the ideal approach in regard to BC screening should be personalized, based on each woman’s personal needs, comorbidities, functionality, and life expectancy [30,31,32]. 

Regional differences in BC incidence have been investigated over time, with interesting findings so far. One study investigating the incidence of triple-negative BC among non-Hispanic Black (NHB) women in the US from 2011 to 2015 found that this population is more susceptible to developing the disease. Significant differences were noted between the studied regions (South, Midwest, West, and North-East); these differences might be explained by genetic admixture among people with different geographic ancestral origins, emphasizing a personalized approach in different geographical regions of a country/in different states [33]. 

The primary purpose of this study is to provide more in-depth insight into various demographic, clinical, and lifestyle factors in relation to BC and to predict the extent to which certain variables described as “predictors” might lead to further investigation. By analyzing a large cohort, we are able to provide valuable and up-to-date information on BC screening, support breast specialists, and further enhance international screening guidelines.

## 2. Materials and Methods

### 2.1. Data Collection

This study represents the first organized round of BC screening in Romania, as no national screening program has been previously implemented. All participants were enrolled for their initial screening round during the study period (July 2020 to December 2023).

This study retrospectively registered and analyzed anonymized data from a cohort of 58,760 patients aged 50–69, with no history of breast malignancies who underwent BC screening in the West (W), North-East (NE), North-West (NW), and South-East (SE) regions of Romania from July 2020 to December 2023. The screening process was carried out in a multiphase manner which consisted of three distinct stages. The first phase of the screening process was conducted using 2D mammography as the primary screening method. In the second stage of the screening process, breast ultrasound was performed as a supplemental imaging tool in specific cases of a positive (or inconclusive) mammographic result. Depending on the case, spot compression (focal compression) or 3D mammography were applied in certain instances. The criteria for determining whether ultrasound was necessary included the presence of dense breast tissue, suspicious findings on mammography, and clinical indications such as palpable lumps or a high-risk personal or familial history of breast cancer. The last phase of the screening process was represented by echographic-guided biopsy in patients who received a positive echographic result.

The cases that presented suspicious microcalcifications on mammographic examination, but with no echographic correspondence, were later evaluated for vacuum-assisted breast biopsy under mammographic guidance based on radiological criteria, following the current clinical guidelines. The statistical analysis in this study used positive and negative variables, focusing on patients who progressed through the screening stages based on both mammographic and ultrasound findings. No stratification by risk category was applied, as all participants were considered part of the general screening population.

The numerical variables included age, BMI, and reproductive factors such as age at menarche, age at first birth, and age at menopause. Categorical variables examined included a family history of cancer, hormonal treatment during menopause, alcohol consumption, smoking habits, and breastfeeding practices, along with the duration of breastfeeding.

### 2.2. Inclusion and Exclusion Criteria

This study included women aged 50–69 years who participated in BC screening programs in the NE, NW, SE, and W regions of Romania between July 2020 and December 2023. Participants were required to have no prior history of breast malignancies or ongoing treatment for breast cancer. Women with incomplete clinical records, previous breast cancer diagnoses, or a history of bilateral mastectomy were excluded from the analysis.

### 2.3. Statistical Analysis

In this study, a comprehensive statistical analysis was conducted to evaluate predictors and outcomes associated with BC screening, with a focus on understanding the factors influencing progression through the screening stages and the relationships between demographic, clinical, and lifestyle variables. The analysis aimed to identify significant differences between subgroups, evaluate progression from mammography to echography and biopsy, and explore interactions between key predictors, such as age and BMI, on screening outcomes.

Continuous variables were summarized using medians and interquartile ranges (Q25–Q75), reflecting the non-normal distribution of most data. Categorical variables were described using counts and percentages. To test for differences between subgroups, non-parametric tests were employed. Specifically, the Mann–Whitney U test was used for the pairwise comparisons of continuous variables, while the Kruskal–Wallis test was applied for comparisons across multiple groups. For categorical variables, the Pearson Chi-Squared test was used to assess differences in proportions, ensuring the robust statistical evaluation of associations in large sample sizes.

To evaluate the progression of patients through the screening stages, logistic regression models were employed. These models identified significant predictors of progression from mammography to echography and from echography to biopsy, providing insights into the factors influencing diagnostic referral decisions. Odds ratios (OR) with 95% confidence intervals (CIs) were reported for significant predictors, and Nagelkerke R^2^ values were calculated to assess the explanatory power of the models.

Interaction analyses were conducted to explore the combined effect of predictors, such as age and BMI, on the likelihood of positive mammography results. These analyses involved the interaction terms in logistic regression models, with the results being visualized using interaction plots to illustrate how the relationship between predictors and outcomes varied across levels of key variables.

All statistical analyses were conducted at a significance threshold of *p* < 0.05. The statistical software R (version 4.3.0; R Core Team, 2023) and RStudio (version 2023.06.0+421; RStudio Team, 2023) were used for all computations and visualizations, ensuring reproducibility and rigor. Visual representations, including violin plots, bar charts, and interaction plots, were utilized to enhance the interpretability of findings. These analyses provide a comprehensive understanding of the predictors and outcomes associated with BC screening, emphasizing key demographic and clinical factors in guiding diagnostic progression and stratification.

## 3. Results

The study analyzed a cohort of 58,760 patients to assess various demographic, clinical, and lifestyle variables in relation to breast cancer screening. Numerical variables included age, BMI, and reproductive factors such as age at menarche, age at first birth, and age at menopause. BMIs were calculated as weight in kilograms divided by height in meters squared (kg/m^2^) and was categorized based on the World Health Organization (WHO) guidelines into underweight, normal weight, overweight, and obese groups. Age at menarche was defined as the self-reported age at which menstrual periods began, while age at first birth and age at menopause were based on patient-reported data reflecting key reproductive milestones with potential implications for breast cancer risk due to hormonal exposure.

Categorical variables included a family history of cancer, hormonal treatment during menopause, alcohol consumption, smoking habits, and breastfeeding practices, along with the duration of breastfeeding. A family history of cancer was defined as the presence of breast or other cancers among first-degree relatives (parents, siblings, or children). Hormonal treatment during menopause referred to the use of hormone replacement therapy (HRT) for managing menopausal symptoms, as recommended by clinical guidelines. HRT is known to influence BC risk due to its impact on prolonged exposure to estrogen. Alcohol consumption was defined as the self-reported intake of any alcoholic beverages, with its role in BC being linked to increased levels of circulating estrogen and carcinogenic metabolites. Smoking was assessed based on self-reported tobacco use, categorized as current smokers, occasional smokers, or non-smokers, due to its potential carcinogenic effects.

Daily physical activity (DPA) was defined according to the WHO guidelines as engaging in moderate to vigorous activity, such as walking, cycling, or sports, for at least 150 min per week. Physical activity is known to reduce breast cancer risk by modulating hormonal levels, improving immune function, and maintaining a healthy weight. Breastfeeding practices were categorized into durations (<1 month, 1–6 months, 6–12 months, and >12 months) based on self-reported data. Breastfeeding is recognized for its protective effect against BC through hormonal changes that reduce lifetime estrogen exposure.

The study analyzed several numerical variables related to participant demographics and reproductive health. These variables were summarized using medians and interquartile ranges (Q25–Q75), providing an accurate representation of their distribution within the study population.

The median age of participants was 56 years, with an interquartile range of 53 to 62 years, indicating that most participants were middle-aged. BMI values ranged from 25.00 to 31.60 kg/m^2^, with a median of 28.12 kg/m^2^, reflecting a tendency toward the overweight category among the participants.

Reproductive health-related variables showed that the median age at menarche was 14 years (Q25–Q75: 13–14) and the median age at first birth was 22 years, with an interquartile range of 20 to 25 years. The age at menopause had a median value of 49 years, with an interquartile range from 46 to 51 years, suggesting a relatively consistent onset of menopause among the participants. The results are presented in Table 1.

Several categorical variables were analyzed to capture key participant characteristics related to familial history, lifestyle factors, reproductive history, and breastfeeding practices. The results are presented as frequencies and proportions in Table 2.

A small proportion of participants reported having an FMH of cancer, with 6.19% (n = 3638) indicating a positive response. Similarly, 6.04% (n = 3548) of participants reported undergoing hormonal treatment during menopause. Alcohol consumption was relatively uncommon, reported by 5.16% (n = 3032) of participants, while smoking was more prevalent, with 16.05% (n = 9431) identifying as smokers.

Breastfeeding practices were widespread, with 93.64% (n = 55,024) of participants reporting that they had breastfed. Among those who breastfed, the duration varied widely: 4.17% (n = 2450) breastfed for less than one month, 44.55% (n = 26,176) for less than six months, 30.14% (n = 17,711) for six to twelve months, and 21.14% (n = 12,423) for more than twelve months.

Reproductive history showed that most participants had at least one birth, with 6.94% (n = 4076) reporting no births, 31.43% (n = 18,468) reporting one birth, 44.62% (n = 26,218) reporting two births, and 17.01% (n = 9998) reporting three or more births.

A notable proportion of participants (32.36%, n = 19,016) reported engaging in DPA, reflecting lifestyle patterns within the cohort. Additionally, 43.69% (n = 25,672) were identified as vulnerable (as per the study criteria).

### 3.1. Screening Process and Patient Progression Through Diagnostic Stages

The analysis of patient flow through the BC screening stages provides an insightful overview of the structured diagnostic pathway, highlighting the progressive narrowing of patient numbers at each stage. All 58,760 patients initiated the process with mammography, the primary screening tool. Of these, 3197 patients (5.44%) received a positive mammography result (MP) and were referred for additional testing via echography. Among those undergoing echography, 688 patients (21.52%) proceeded to biopsy based on positive echography findings (EP). This sequential reduction in patients reflects the effectiveness of the multi-step screening process in filtering individuals who require further diagnostic evaluation.

**Figure 1:**
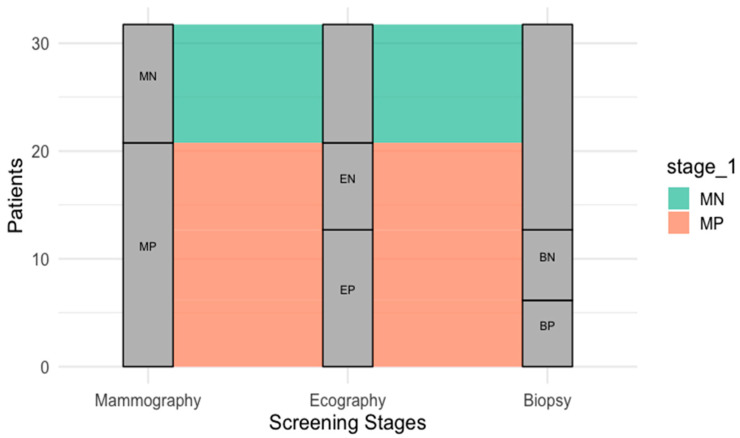
Patient flow through BC screening stages. This flowchart illustrates the progressive reduction in patient numbers through the three stages of breast cancer screening: mammography, echography (complementary imaging modality), and biopsy. Of the 58,760 women screened with mammography, 3197 (5.44%) had a positive result and were referred for echography. Of these, 688 patients (21.52%) progressed to biopsy. Positive and negative results are shown for each stage to depict the filtering effect of the screening process. MP—mammography positive. MN—mammography negative. EN—echography negative. EP—echography positive. BN—biopsy negative. BP—biopsy positive.

The bar chart in Figure 1 illustrates this patient progression across the screening stages, using log-transformed patient counts to emphasize the relative proportions advancing through each phase. The chart highlights the substantial reduction in patient numbers from mammography to echography, reflecting the low percentage of positive initial findings (5.44%). The subsequent transition from echography to biopsy shows an even more concentrated selection, with 21.52% of patients tested at the echography stage advancing to biopsy.

The low recall rate of 5.44% observed in our cohort is lower than the recall rates reported in other European countries, which range between 7% and 10%. This may be attributed to the specific particularities of the screened population, including lower breast density in the predominantly postmenopausal cohort, rural–urban disparities in breast composition, or differences in mammographic interpretation protocols. Further analysis is needed to evaluate the potential impact of false negatives, as follow-up data on interval cancers were not included in this study.

This patient flow underscores the precision of the breast cancer screening process. The narrowing funnel reflects the stepwise approach designed to identify patients most at risk while minimizing unnecessary diagnostic procedures for those with negative findings. The distribution of positive and negative results across each stage (e.g., MN-mammographic negative vs. MP, EN-echographic negative vs. EP, BN-biopsy negative vs. BP-biopsy positive) aligns with the expected outcomes in large-scale screening programs, ensuring a balance between thorough investigation and resource efficiency.

### 3.2. Rural vs. Urban Differences in Breast Cancer Screening in Romania

The analysis of rural versus urban populations in the context of breast cancer screening revealed notable differences across a range of demographic, reproductive, lifestyle, and screening-related variables. These disparities provide critical insights into the epidemiological patterns associated with BC screening outcomes.

The median age of participants was slightly lower in the rural group (56 years; interquartile range [IQR]: 53–61) compared to the urban group (57 years; IQR: 53–62), a difference that was statistically significant (*p* < 0.001). The BMI was also significantly higher in the rural group, with a median of 28.58 kg/m^2^ (IQR: 25.51–32.03) versus 27.73 kg/m^2^ (IQR: 24.77–31.25) in the urban group (*p* < 0.001). While the median age at menopause was consistent at 49 years for both groups, the interquartile range for urban participants was slightly wider (46–51) compared to rural participants (46–50), a statistically significant difference (*p* < 0.001). Similarly, the age at first birth differed significantly, with rural participants having a median of 21 years (IQR: 20–24) compared to 22 years (IQR: 21–26) for urban participants (*p* < 0.001). The age at menarche showed a small but statistically significant difference, with rural participants reporting a median of 14 years (IQR: 13–15) compared to 14 years (IQR: 13–14) for urban participants (*p* < 0.001). The results are presented in Table 3.

The prevalence of vulnerability was overwhelmingly higher in rural areas, with 99.89% of rural participants classified as vulnerable compared to only 3.48% in urban areas (*p* < 0.001). Breastfeeding practices were widespread in both groups but slightly more common in rural participants (94.04%) compared to urban participants (93.36%; *p* < 0.001). However, significant differences emerged in the distribution of the number of births. Rural participants were more likely to have three or more children (26.36% vs. 10.33%), while urban participants were more likely to have no children (8.32% vs. 5.01%) or one child (37.62% vs. 22.78%; *p* < 0.001). Similarly, breastfeeding duration differed significantly, with rural participants more likely to breastfeed for over 12 months (27.65% vs. 16.48%), while urban participants were more likely to breastfeed for shorter durations (*p* < 0.001).

Lifestyle factors showed notable urban–rural differences. DPA was more common in rural participants (34.87%) compared to urban participants (30.57%; *p* < 0.001). Smoking was significantly more prevalent among urban participants (18.03%) than rural participants (13.29%; *p* < 0.001). Alcohol consumption, though low overall, was slightly more common in rural areas (5.53% vs. 4.9%; *p* < 0.001). A higher proportion of urban participants reported an FMH of cancer (6.8%) compared to rural participants (5.34%; *p* < 0.001). Similarly, hormonal treatment during menopause was slightly more prevalent in urban participants (6.55%) compared to rural participants (5.32%; *p* < 0.001). The results are presented in Table 4.

The positivity rate for mammography results was significantly higher in urban participants (7.09%) compared to rural participants (5.5%; *p* < 0.001). This suggests that urban participants were more likely to have suspicious findings on initial BC screening, potentially reflecting differences in risk factors or healthcare access. The results are presented in Table 5.

Among participants who underwent echography following a positive mammography, the positivity rates were slightly higher in the rural group (29.56%) compared to the urban group (27.91%; *p* < 0.001). Despite the small numerical difference, the statistical significance indicates that rural participants might experience a higher likelihood of suspicious findings on follow-up imaging. The results are presented in Table 6.

Among the 58,760 participants, a total of 688 biopsies were performed following a positive echography, yielding 470 positive biopsy results. This corresponds to a detection rate of 8 per 1000 patients. Among those who underwent biopsy, the positivity rates were higher in urban participants (70.81%) compared to rural participants (64.03%). However, this difference was not statistically significant (*p* = 0.07), suggesting that the biopsy confirmation rates were broadly similar across groups, albeit slightly higher in urban areas. The results are presented in Table 7.

### 3.3. Regional Differences in Breast Cancer Screening in Romania

The stratified analysis by region provides detailed insights into the demographic, reproductive, lifestyle, and BC screening characteristics across four major regions of Romania: NE, NW, SE, and W. This regional analysis highlights significant disparities in the variables of interest and their potential epidemiological implications for BC screening outcomes.

Significant regional differences were observed in participants’ age, BMI, and reproductive factors. Median age was slightly higher in the NV and W regions (both 57 years; IQR: 53–62) compared to the NE and SE regions (both 56 years; IQR: 53–62; *p* < 0.001). The BMI also varied significantly, with the NE region reporting the highest median BMI (28.34 kg/m^2^; IQR: 25.34–31.64) and the SE region the lowest (27.68 kg/m^2^; IQR: 24.78–31.24; *p* < 0.001). Age at menopause and age at menarche showed slight but statistically significant regional variations, with a consistent median of 49 years for menopause and 14 years for menarche across all regions, differing mainly in interquartile ranges. The median age at first birth ranged from 22 years in the NE, NW, and SE regions to 22 years (IQR: 21–26) in the W region, highlighting later childbearing trends in the West (*p* < 0.001). The results are presented in Table 8.

The prevalence of vulnerability was highest in the SE region (63.28%), followed by NE (50.77%) and NW (37.42%), and was lowest in the W region (29.83%; *p* < 0.001). Breastfeeding practices were widespread across all regions, ranging from 94.17% in the NE to 92.98% in the W region (*p* < 0.001). However, breastfeeding duration varied significantly. Longer breastfeeding periods (>12 months) were most common in the SE region (28.86%) and NE region (26.07%), whereas shorter durations (1–6 months) were more prevalent in the W (55.11%) and NW (48.27%) regions (*p* < 0.001).

Reproductive patterns also varied. The proportion of participants with three or more births was highest in the NE region (22.84%) and lowest in the W region (9.92%; *p* < 0.001). Conversely, the W region reported the highest proportion of participants with only one birth (43.58%) compared to other regions. Lifestyle factors showed notable disparities. Daily physical activity (DPA) was most common in the W region (51.82%) and least common in the SE (28.48%) and NE (28.76%) regions (*p* < 0.001). Smoking prevalence was highest in the W region (19.49%) and NW region (18.19%), with the NE and SE regions reporting lower rates (12.12% and 13.17%, respectively; *p* < 0.001). Alcohol consumption exhibited stark regional differences, being notably higher in the NE (12.66%) compared to other regions, where the rates were below 9% (*p* < 0.001). A family medical history (FMH) of cancer was most frequently reported in the W (7.21%) and NE (7.02%) regions (*p* < 0.001). The results are presented in Table 9.

The proportion of positive mammography results varied significantly by region, ranging from 5.46% in the SE region to 10.08% in the W region (*p* < 0.001). The higher positivity rate in the W region may reflect increased healthcare access or potentially higher risk factors among participants in this region. The results are presented in Table 10.

Among the participants who underwent echography following a positive mammography, the positivity rates were significantly different across regions. The NE region reported a positivity rate of 27.87%, similar to NV (30.96%) and W (30.65%), whereas the SE region had a markedly lower rate of 14.09% (*p* < 0.001). These differences may reflect variations in diagnostic follow-up or criteria for referral to echography. The results are presented in Table 11.

The biopsy positivity rates showed no statistically significant regional differences (*p* = 0.22). The positivity rates were highest in the NE region (71.98%), followed by the W (69.16%) and NW (67.16%) regions, while the SE region had the lowest rate (54.55%). The lack of statistical significance suggests broadly similar biopsy confirmation rates across regions, despite differences in mammography and echography positivity. The results are presented in Table 12.

### 3.4. Stratified Analysis by Mammography Positivity

The stratified analysis based on mammography positivity offers a detailed comparison between participants with positive mammography results and those with negative results. This analysis sheds light on the demographic, reproductive, and lifestyle factors associated with screening outcomes, providing valuable insights into potential risk factors for breast cancer detection.

Participants with positive mammography results were found to have slightly different characteristics compared to those with negative results. While the median age was consistent across both groups at 56 years, the interquartile range showed minor differences, and the *p*-value (0.02) indicates statistical significance. The BMI was significantly lower in the positive mammography group (median: 27.43 kg/m^2^; IQR: 24.38–31.14) compared to the negative group (median: 28.12 kg/m^2^; IQR: 25.07–31.63; *p* < 0.001). Similarly, age at menopause was marginally later for the positive group (median: 49 years; IQR: 47–51) compared to the negative group (median: 49 years; IQR: 46–51; *p* < 0.001). Age at first birth was slightly higher in the positive group (median: 22 years; IQR: 21–25) compared to the negative group (median: 22 years; IQR: 20–25; *p* < 0.001). No significant difference was observed for age at menarche (*p* = 0.16), suggesting that this variable was not associated with mammography positivity. The results are presented in Table 13.

Several categorical variables showed significant differences between the two groups. Vulnerability was less prevalent among those with positive MP (37.65%) compared to the negative group (44.1%; *p* < 0.001). Breastfeeding was slightly less common in the positive group (92.69%) compared to the negative group (93.71%; *p* = 0.01), although the absolute difference was minimal. Notable differences emerged in the distribution of the number of births. Participants with positive mammography results were more likely to have had no births (9.74%) or only one birth (34.63%) compared to the negative group (6.74% and 31.21%, respectively). Conversely, those with negative results were more likely to have had three or more births (17.3%) compared to the positive group (12.92%; *p* < 0.001).

Breastfeeding duration also differed between the groups. Shorter breastfeeding durations (1–6 months) were more common in the positive group (47.97%) compared to the negative group (44.31%), whereas longer durations (>12 months) were less common in the positive group (18.4%) compared to the negative group (21.33%; *p* < 0.001). Lifestyle factors showed mixed associations. Smoking was more prevalent among those with positive mammography results (18.14%) compared to those with negative results (15.91%; *p* < 0.001). FMH was also significantly more common in the positive group (7.7%) than in the negative group (6.09%; *p* < 0.001). However, DPA, alcohol consumption, and hormonal treatment during menopause showed no significant differences between the two groups. The results are presented in Table 14.

### 3.5. Stratified Analysis by Age Groups

This stratified analysis examines key demographic, reproductive, lifestyle, and breast cancer screening characteristics across three age groups: under 50 years, 50–60 years, and over 60 years. The results highlight significant variations across age groups, shedding light on age-related differences in risk factors and screening outcomes.

The BMI increased with age, with the youngest group (<50 years) having the lowest median BMI (26.99 kg/m^2^; IQR: 24.02–30.42) compared to 50–60 years (27.92 kg/m^2^; IQR: 24.88–31.53) and >60 years (28.67 kg/m^2^; IQR: 25.64–32.02; *p* < 0.001). Age at menopause was consistent across groups, with a median of 49 years, but the interquartile range widened in the >60 group (45–52) compared to younger groups, indicating greater variability (*p* < 0.001). Age at first birth showed a decreasing trend with increasing age, with younger participants having a median age at first birth of 23 years (IQR: 21–27) compared to 22 years (IQR: 20–25) for the 50–60 group and 22 years (IQR: 20–24) for the >60 group (*p* < 0.001). Age at menarche was consistent across groups, with a median of 14 years and slight variations in the interquartile ranges (*p* < 0.001). The results are presented in Table 15.

The proportion of participants classified as vulnerable decreased with age, from 48.7% in the <50 group to 45.04% in the 50–60 group and 39.88% in the >60 group (*p* < 0.001). Breastfeeding was widespread across all groups but slightly more prevalent in older participants (>60 years: 94.02%) compared to the youngest group (<50 years: 92.68%; *p* = 0.004). The distribution of the number of births showed significant differences, with the youngest group being more likely to have no births (8.43%) or one birth (36.81%) compared to the oldest group (>60 years), where 23.57% had three or more births (*p* < 0.001).

Breastfeeding duration also varied significantly. Longer breastfeeding periods (>12 months) were more common in the youngest group (24.02%) compared to the >60 group (20.45%), while shorter durations (1–6 months) were slightly more common in the older groups (*p* < 0.001). Hormonal treatment during menopause was most prevalent in the 50–60 group (6.18%) compared to the <50 group (4.88%; *p* = 0.005). Smoking prevalence decreased with age, being highest in the <50 group (19.11%) and lowest in the >60 group (12.13%; *p* < 0.001). A family medical history of cancer was more frequently reported in the >60 group (6.83%) compared to the <50 group (6.06%) and 50–60 group (5.89%; *p* < 0.001). The results are presented in Table 16.

The mammography positivity rates differed significantly by age group. The youngest group (<50 years) had the highest positivity rate (7.27%), compared to 6.35% in the 50–60 group and 6.41% in the >60 group (*p* < 0.001). This trend suggests that younger participants who undergo mammography may represent a higher risk subgroup, warranting further investigation. The results are presented in Table 17.

Among participants referred for echography after a positive mammography result, the positivity rates increased significantly with age. The youngest group had the lowest echography positivity rate (22%), compared to 23.3% in the 50–60 group and 40.53% in the >60 group (*p* < 0.001). This indicates that older participants with positive mammography results were more likely to have suspicious findings on follow-up imaging. The results are presented in Table 18.

The BP rates also increased significantly with age. The youngest group had the lowest biopsy positivity rate (46.15%), compared to 64.04% in the 50–60 group and 75.9% in the >60 group (*p* < 0.001). These results suggest that older participants with suspicious findings on imaging are more likely to receive a definitive diagnosis of breast cancer following biopsy. The results are presented in Table 19.

### 3.6. Stratified Analysis by BMI Groups

This analysis examines participants across four BMI categories: underweight, normal weight, overweight, and obese. Stratification by BMI provides insights into how weight status influences demographic, reproductive, lifestyle, and breast cancer screening variables.

Significant differences in age, reproductive variables, and age at menarche were observed across BMI categories. Participants in higher BMI groups tended to be older, with the median age increasing from 55 years in the underweight and normal weight groups to 57 years in the obese group (*p* < 0.001). Age at menopause showed a similar trend, with the median increasing from 48.5 years in the underweight group to 49 years in the overweight and obese groups (*p* < 0.001). Age at first birth decreased slightly with increasing BMI, with the underweight group having the highest median age of 22 years (IQR: 20–26), compared to 22 years (IQR: 20–24) in the obese group (*p* < 0.001). Age at menarche was largely consistent across all groups, with a median of 14 years (*p* < 0.001), indicating a limited association with BMI. The results are presented in Table 20.

The prevalence of vulnerability increased with BMI, from 44.97% in the underweight group to 48.02% in the obese group (*p* < 0.001). Breastfeeding was slightly less common among underweight participants (92.6%) compared to overweight (94.03%) and normal weight (93.75%) participants (*p* = 0.001). The number of births also varied significantly, with the proportion of participants having three or more births increasing from 15.68% in the underweight group to 20.83% in the obese group (*p* < 0.001). Conversely, the proportion of participants with no births decreased with increasing BMI.

Breastfeeding duration showed notable differences across BMI categories. The underweight group had the highest proportion of participants breastfeeding for 1–6 months (54.73%) compared to obese participants (43.17%; *p* < 0.001). Longer breastfeeding periods (>12 months) were more common in obese participants (23%) compared to the underweight group (19.53%). Daily physical activity (DPA) decreased with increasing BMI, from 39.94% in the underweight group to 26.97% in the obese group (*p* < 0.001). Smoking prevalence also declined with increasing BMI, with the highest rates observed in the underweight group (35.21%) and the lowest in the obese group (12.91%; *p* < 0.001). Other variables, such as alcohol consumption and a family history of cancer, showed no significant differences across BMI categories. The results are presented in Table 21.

The MP rates decreased as the BMI increased. The underweight group had the highest positivity rate (8.58%), followed by the normal weight (7.76%), overweight (6.3%), and obese (5.62%) groups (*p* < 0.001). This trend suggests a potential inverse association between BMI and mammography positivity, possibly reflecting differences in breast density or diagnostic challenges in higher BMI groups. The results are presented in (Table 22.)

Among the participants referred for echography, the positivity rates increased with BMI. The underweight group had a positivity rate of 31.58%, while the obese group had the highest rate, at 36.88% (*p* < 0.001). This finding contrasts with the mammography results, suggesting that follow-up imaging is more likely to confirm suspicious findings in higher BMI groups. The results are presented in Table 23.

The biopsy positivity rates did not show statistically significant differences across BMI groups (*p* = 0.16). However, all biopsies in the underweight group were positive (100%), though this group had the smallest sample size (n = 4). The positivity rates ranged from 68.18% in the normal weight group to 71.38% in the obese group, indicating consistent diagnostic confirmation rates across BMI categories. The results are presented in Table 24.

### 3.7. Predictors of Progression to Echography

The logistic regression analysis aimed to identify significant predictors for progression from mammography to echography, providing insights into the factors influencing referral decisions following an initial mammography. While the Nagelkerke R^2^ value of 0.004 indicates that the model explains a small proportion of the variance, the findings highlight key patient characteristics that contribute to this diagnostic pathway. These results, though limited in scope, underscore the multifaceted nature of referral decisions and the need to consider a broad range of factors in future models.

BMI emerged as a significant predictor, with a slight but statistically significant association with reduced odds of progression to echography (OR: 0.98; 95% CI: 0.98–0.99, *p* < 0.001). This finding suggests that for each unit increase in BMI, the likelihood of referral decreases marginally. Although the effect size is small, this result raises important considerations.

A FMH was strongly associated with progression to echography, with patients reporting aa FMH having a 28% higher likelihood of referral compared to those without a family history (OR: 1.28; 95% CI: 1.11–1.46, *p* < 0.001). This highlights the importance of familial risk factors in clinical decision-making, consistent with established guidelines that prioritize additional diagnostic evaluations for individuals with hereditary risk. The results emphasize the role of a FMH as a critical component in assessing breast cancer risk and guiding appropriate follow-up.

Reproductive factors also significantly influenced referral patterns. Age at first birth was positively associated with progression to echography, with the odds of referral increasing by 3% for each additional year of age at first birth (OR: 1.03; 95% CI: 1.02–1.03, *p* < 0.001). Similarly, age at menopause was positively associated with referral, with a 2% increase in the odds of progression for each additional year of age at menopause (OR: 1.02; 95% CI: 1.01–1.02, *p* < 0.001). Both findings align with established breast cancer risk profiles. A later age at first birth and later menopause are well-documented risk factors, reflecting the extended exposure to estrogen and other hormonal influences that increase susceptibility to BC. The results suggest that clinicians are appropriately factoring these reproductive characteristics into their referral decisions.

While the predictors identified by the model are statistically significant and clinically relevant, the low Nagelkerke R^2^ value underscores the complexity of referral decisions. Factors not captured in this model, such as radiological findings, patient symptoms, and clinician judgment, likely play critical roles in determining progression to echography. These findings suggest that while BMI, family history, and reproductive factors provide meaningful insights, a more comprehensive approach incorporating additional variables is needed to better understand the screening process. This analysis offers a foundation for refining diagnostic protocols and addressing disparities in follow-up care, particularly for high-risk populations such as those with a higher BMI or significant familial risk factors. The results are detailed in Table 25.

### 3.8. Predictors of Progression to Biopsy

The logistic regression analysis to identify predictors of progression from echography to biopsy highlights the key patient characteristics influencing the likelihood of further diagnostic evaluation. While the model’s Nagelkerke R^2^ value of 0.06 indicates that it explains 6% of the variance, a modest improvement over previous models, the findings provide meaningful insights into factors guiding clinical decisions.

Age emerged as a strong predictor of progression to biopsy, with an odds ratio (OR) of 1.08 (95% CI: 1.05–1.11, *p* < 0.001). This indicates that for each additional year of age, the likelihood of being referred for biopsy increased by 8%. This finding aligns with the established role of age as a significant risk factor for BC, with older patients at greater risk of developing malignancies. Consequently, clinicians may be more likely to recommend biopsy for older patients following abnormal echography results to rule out cancerous lesions.

Age at first birth also significantly influenced biopsy referrals, with an OR of 1.04 (95% CI: 1.00–1.08, *p* < 0.001). This suggests that women who had their first child later in life were slightly more likely to progress to biopsy, with the odds increasing by 4% for each additional year of age at first childbirth. Later childbirth is a known risk factor for breast cancer, as it delays the protective hormonal changes associated with pregnancy. This result indicates that reproductive history is appropriately factored into clinical decisions for additional diagnostic evaluation.

The BMI was not a significant predictor in this analysis (OR = 1.02, 95% CI: 0.99–1.05, *p* = 0.286), suggesting that the BMI did not meaningfully influence the likelihood of progression to biopsy after echography. This contrasts with the BMI’s role in earlier stages of the screening process and implies that once an abnormality is detected on echography, other factors, such as imaging findings and clinical suspicion, take precedence over weight-related considerations in determining the need for a biopsy.

Although the model identified significant associations with age and reproductive history, its relatively low Nagelkerke R^2^ value underscores the complexity of referral decisions for biopsy. Factors not included in the model, such as echography findings, clinician judgment, and patient symptoms, are likely to play substantial roles in determining progression to biopsy. These results highlight the importance of considering both measurable patient characteristics and unmeasured clinical factors in understanding this diagnostic pathway. The detailed results of this analysis are presented in Table 26.

### 3.9. Interaction Analysis Between Age and BMI on Mammography Positivity

This analysis explores the interaction between age and BMI in predicting the likelihood of a positive mammography result. By examining how the relationship between age and mammography positivity changes across different levels of BMI, this analysis provides a deeper understanding of how these variables jointly influence screening outcomes.

**Figure 2:**
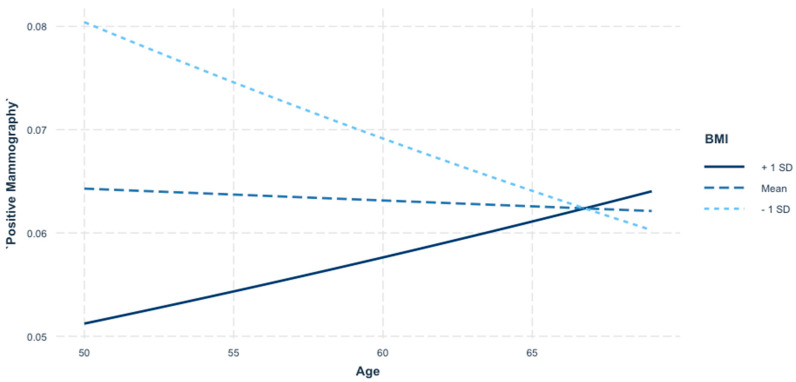
Interaction between age and BMI in predicting positive mammography results. The interaction plot illustrates how the likelihood of a positive mammography result changes with age across three BMI categories: one standard deviation (SD) below the mean BMI (−1 SD), mean BMI, and one SD above the mean BMI (+1 SD). Younger individuals with a lower BMI have a higher probability of positive results, whereas the likelihood increases for older individuals with a higher BMI, suggesting distinct risk patterns across subgroups.

The interaction plot presented in Figure 2 illustrates how the likelihood of a positive mammography result varies by age, stratified across three BMI categories: one standard deviation (SD) above the mean (+1 SD), the mean BMI, and one SD below the mean (−1 SD). The results indicate that age interacts with BMI to influence mammography positivity, revealing distinct trends across BMI categories.

For individuals with a low BMI (−1 SD), the probability of a positive mammography result decreases with age. Conversely, for individuals with an average BMI, the probability remains relatively stable across ages, with only a slight upward trend. However, among individuals with a high BMI (+1 SD), the probability of a positive mammography result increases significantly with age.

## 4. Discussions

### 4.1. Rural vs. Urban Differences

The results demonstrate significant rural–urban disparities in demographic, reproductive, lifestyle, and breast cancer screening variables. Rural participants tended to be younger, had higher BMIs, and reported earlier ages at first birth and menarche. Vulnerability was overwhelmingly higher in rural areas, likely reflecting socioeconomic or healthcare access disparities. Rural participants also had more children and were more likely to breastfeed for longer durations, reflecting traditional reproductive and childcare practices. One study analyzing disparities between rural and urban populations concluded that, although breast cancer incidence is lower on average in rural areas, the breast cancer mortality rates are comparable [34]. 

Lifestyle differences revealed that urban participants were more likely to smoke but less likely to engage in daily physical activity, potentially reflecting differences in occupational and environmental factors. Urban participants also had higher rates of family history of cancer and hormonal treatment during menopause, which may correlate with increased healthcare access or awareness [35]. 

We compared positive cases across the rural vs. urban environment with interesting yet somehow predictable results. Our findings stated that urban participants were more likely to have positive mammography results (2430 positive cases vs. 1347 in rural area), as well as echographic results (596 positive cases vs. 314 in rural areas). Lastly, when referring to biopsy rates, the urban environment maintains a higher trend, with 308 positive cases vs. 162 in rural areas. The results are noteworthy and align with the current literature, which indicates that patients located in the urban setting have better access to mammography centers and healthcare services in general but also tend to display decreased physical activity and unhealthy habits, such as smoking [36]. 

The detection rate reported from our study was found to be 8 per 1000 patients, meaning that out of the 58,760 participants in this study, 470 patients were categorized positive through biopsy. These results are in agreement with established European screening programs such as the German mammographic screening program(MSP), which reported a total breast cancer detection rate of 8.1/1000 patients during the 2005–2009 timeline [37]. 

### 4.2. Regional Differences

The regional analysis reveals striking disparities in demographic, reproductive, lifestyle, and breast cancer screening variables across Romania. The NE and SE regions showed higher vulnerability rates, reflecting socioeconomic disparities that may influence healthcare access and outcomes. Breastfeeding practices and longer breastfeeding durations were more common in the NE and SE regions, likely reflecting traditional child-rearing practices in these areas. Breastfeeding is a known protective factor against BC disease in various literature studies [21]. 

Lifestyle factors, such as physical activity, smoking, and alcohol consumption, exhibited substantial regional differences. The high prevalence of smoking and alcohol consumption in the NE region raises public health concerns, while the W region reported the highest rates of daily physical activity, likely influenced by cultural or occupational patterns.

Screening outcomes also varied significantly. The W region had the highest mammography positivity rate, potentially indicating more effective screening or higher BC risk. However, the SE region had notably lower echography positivity rates, suggesting differences in follow-up protocols or access to diagnostic services. Despite these differences, the biopsy confirmation rates were comparable across regions, indicating consistency in diagnostic accuracy. Our results extend the current information regarding possible correlations between country region and the rate of positive mammographies/breast ultrasounds, the amount of daily physical activity, or habits such as alcohol intake or smoking [38]. 

While this study focused on Romania, the regional disparities observed, such as differences in the mammography positivity rates, healthcare access, and lifestyle factors, are not unique to this population. Many countries with regional socioeconomic and healthcare access inequalities may exhibit similar patterns.

### 4.3. Stratified Analysis (Mammography Positivity)

The stratified analysis reveals distinct differences between participants with positive and negative mammography results, highlighting key demographic, reproductive, and lifestyle factors that may be associated with an increased likelihood of mammography positivity.

A lower BMI among participants with positive results may reflect underlying metabolic or hormonal factors associated with breast cancer risk. The slightly later age at menopause in the positive group is consistent with the existing literature, linking extended estrogen exposure to increased breast cancer risk. Similarly, the higher prevalence of having no or only one birth in the positive group aligns with reproductive patterns associated with increased breast cancer risk, likely due to the reduced protective effects of pregnancy and breastfeeding.

Lifestyle factors, including smoking and a family history of cancer, emerged as important contributors. The higher smoking prevalence among participants with positive results reinforces its role as a modifiable risk factor. The higher prevalence of a family history of cancer among the positive group underscores the importance of genetic predisposition in BC screening outcomes. Modifiable habits or measurements, such as BMI and smoking, and non-modifiable characteristics, such as age at menopause and a positive FMH of breast cancer, have previously been described in other studies as well as key factors linked with mammographic positivity or increased BC risk [33]. 

### 4.4. Stratified Analysis (Age Groups)

The stratified analysis by age groups reveals important age-related differences in BC screening and associated variables. Older participants tended to have higher BMIs, more children, and longer breastfeeding durations, which are consistent with generational differences in reproductive patterns and lifestyle. Smoking prevalence, however, was notably higher in the youngest group, highlighting the importance of targeted public health interventions for this age cohort. Previous research have consistently shown smoking as a risk factor [39]. 

Screening outcomes also varied by age. While the mammography positivity rates were slightly higher in the youngest group, the echography and biopsy positivity rates increased significantly with age. These findings suggest that older participants may represent a higher risk population with a greater likelihood of confirmed BC following diagnostic procedures. Conversely, younger participants with positive mammography results may include more false positives or cases requiring further diagnostic clarity. Searching for similar results in the current literature has been scarce; however, it is a known fact that older age correlates with increased BC risk and therefore requires further investigations [1]. 

### 4.5. Stratified Analysis (BMI Groups)

Stratifying results by BMI reveals complex relationships between weight status and BC screening outcomes. Older age, later menopause, and higher parity were associated with a higher BMI, reflecting known epidemiological trends. Lower daily physical activity and reduced smoking prevalence in higher BMI groups underscore lifestyle differences that may influence overall health outcomes. The obesity–breast cancer link has been studied in various previous studies. One study assessed it in relation to menstrual status and found that obese postmenopausal women have an increased risk of developing BC, while obesity may have a protective effect in premenopausal women. However, this pattern was observed exclusively in the European population [40]. 

Screening outcomes showed an intriguing pattern. The mammography positivity rates were highest in the underweight group and lowest in the obese group, suggesting potential diagnostic challenges in higher BMI groups due to factors like increased breast density or imaging limitations. Conversely, echography positivity increased with BMI, indicating that follow-up imaging may more accurately detect abnormalities in this group. The biopsy positivity rates were consistent across BMI categories, suggesting that once abnormalities are identified, diagnostic accuracy is unaffected by BMI. One interesting study stated that obese women had a more than a 20% increased risk of having false-positive mammography results compared with underweight and normal weight women [41]. 

The patterns identified in this study, such as lower mammography positivity in higher BMI groups and increased the echography positivity rates in the same cohort, may hold relevance for countries with comparable challenges in BC imaging. In populations with high obesity rates or resource-limited imaging facilities, these findings can guide improvements in screening protocols, such as implementing tailored imaging techniques for obese women or combining mammography with ultrasound to reduce diagnostic errors. By addressing specific challenges associated with BMI and imaging, these insights can be translated into broader clinical recommendations for other populations.

### 4.6. Predictors of Progression to Echography

The statistical analysis performed in this study also focused on factors that might contribute to the progression of a patient toward echography (the second stage of the screening program). Interestingly, our results showed that patients with a higher BMI are less likely to be referred for further testing. Patients with a higher BMI are at an elevated risk of BC, but imaging challenges associated with increased breast density in higher BMI populations may complicate radiological interpretations, potentially leading to under-referral. These findings suggest a need to re-evaluate screening protocols for higher BMI populations to ensure equitable access to follow-up diagnostics. Research articles in the current literature suggest that obese women exhibit the highest sensitivity to screening mammography, which may explain the lower observed need for further testing in this category [42]. 

Other significant predictors of progression towards echography were a positive FMH of BC, an older age at first birth, and an older menopausal age. These results emphasize the importance of familial risk factors in the decision-making process for further diagnostic evaluation and a correlation between later childbirth and a higher perceived risk of breast cancer, potentially prompting more cautious follow-up in screening. This result aligns with the understanding that later menopause is a known risk factor for breast cancer due to prolonged estrogen exposure caused by a higher number of menstrual cycles and possibly leading to a higher rate of referrals for additional diagnostic procedures. This model highlights key factors, such as a family history of cancer and reproductive characteristics, which play a meaningful role in this decision-making process. However, while these variables are important contributors, additional factors not included in this model likely also influence the referral decision, providing an opportunity for further investigation to improve our understanding of the screening process [4]. 

### 4.7. Predictors of Progression to Biopsy

When investigating factors that could predict further investigation through biopsy in our cohort, older age and older age at first birth were described as statistically relevant predictors. Our results suggest that for each additional year in a patient’s age, the likelihood of progressing from echography to biopsy increased by 8%. Older age is a known risk factor for breast cancer, which may explain why older patients are more likely to be referred for biopsy after an abnormal echography result. Women who had their first child at an older age were slightly more likely to be referred for biopsy, with the odds increasing by 4% for each additional year in age at first birth. This finding aligns with existing evidence suggesting that later childbirth is associated with an increased risk of BC, potentially influencing clinical decisions regarding further diagnostic testing. Although BMI was evaluated in this cohort, it did not significantly impact the likelihood of progression to biopsy after echography, indicating that weight-related factors may not be heavily considered when determining the need for biopsy after an abnormal echography. These results show that while the model explains more variance than the previous analysis, it still captures only a small portion (6%) of the factors influencing progression from echography to biopsy. This suggests that while age and age at first birth are important, additional unmeasured factors likely contribute to the decision to perform a biopsy, and further research is needed to identify these influences [4]. 

Moreover, we investigated how a positive FMH of BC could impact the rates of positive mammography and biopsy results. The statistical analysis of our data showed clear differences in the outcomes based on whether patients had a family history of cancer, emphasizing the role of familial risk in diagnostic decision-making. Patients with a family history of cancer were slightly more likely to receive a positive mammography result (7.9% versus 6%), indicating that family history may increase the likelihood of abnormal findings during initial screening. The comparison was statistically significant, reinforcing the importance of family history in BC screening outcomes. This shows that patients with a family history of cancer not only have a higher likelihood of receiving a biopsy after screening, but they are also more likely to have positive biopsy results, reflecting a higher risk of BC in this group. The difference in biopsy outcomes between patients with and without a family history of cancer was not statistically significant in this dataset. This consistent pattern across both diagnostic stages supports the hypothesis that a FMH is an important risk factor that may prompt a more thorough investigation and lead to higher detection rates of breast cancer. In this regard, current studies focus on the genetic testing of newly diagnosed patients with an early-onset family history, which could provide valuable insights for future treatments and research [43].

### 4.8. Interaction Analysis Between Age and BMI on Mammography Positivity

These findings suggest that the combined influence of age and BMI on mammography positivity is complex and non-linear. They emphasize the need for targeted risk stratification in screening programs, particularly for older individuals with a higher BMI, who may benefit from heightened surveillance or alternative imaging modalities. Conversely, the lower likelihood of positive results in older individuals with a lower BMI warrants further investigation to rule out potential underdiagnosis or imaging limitations in this group. This suggests that older individuals with a higher BMI are more likely to receive positive mammography results compared to younger individuals with a high BMI, highlighting a unique risk trajectory for this subgroup. The link between age and BMI in breast cancer screening has been investigated in a couple of previous research articles, our contribution adding valuable new information on this subject [42]. 

The crossing lines in the plot further underscore the importance of considering interactions between age and BMI in mammography outcomes. At younger ages (around 50), individuals with a lower BMI have a higher probability of a positive result compared to those with a higher BMI. However, this relationship reverses with advancing age, as individuals with a higher BMI become increasingly likely to receive a positive mammography result. These conclusions can be explained by the cumulative effect of obesity and its implications concerning the decreased image contrast, motion unsharpness, as well as the presence of a higher percentage of glandular tissue that can both negatively impact the mammographic interpretation. This trend changes with age, when the percentage of glandular tissue eventually decreases and becomes replaced with adipose tissue, with the increased BMI becoming a potentiating factor in this case [44]. 

#### Limitations and Implications of Our Study

The limitations of this study include its retrospective design, which prevents establishing causality, and specific selection criteria, such as differences in access to screening. There is also the potential presence of unaccounted confounding variables, which could affect the accuracy and generalizability of the findings. Also, while the predictors towards echography/biopsy mentioned in our study proved to be of statistic importance, a low Nagelkerke R^2^ value indicates that additional variables not included in the study are likely to play significant parts as well.

On the other hand, the strong suit of this study is represented by a large cohort of patients that offer valuable results through statistical analyzation. Future studies should aim to incorporate these additional variables to provide a more comprehensive view of the factors influencing biopsy decisions.

This study focused exclusively on women aged 50–69, which aligns with the standard guidelines recommending biennial mammography screening for average-risk women within this age range. While this provides valuable insights into the primary target population for BC screening programs, it does not account for younger women who may have a higher risk due to genetic predispositions, such as BRCA1 or BRCA2 mutations, or other risk factors. Future research should incorporate younger women, particularly those with a positive family history of cancer or other high-risk profiles, to provide a more comprehensive understanding of BC screening needs across all age groups. This would allow for a broader application of findings and the further refinement of screening protocols.

This study is a pilot, meaning that not every region of the country was included and could benefit from mammographic screening; however, a standardized BC screening program is certainly a future goal. In countries in which screening programs are not yet established, pilot studies are crucial in order to increase patient addressability and trust, as well as to build a solid network of mammographic units in places where medical care is less accessible. In one study aiming to analyze the sensitivity of the German MSP, the results varied from 69,9% to 71%, this matter elevating the large-scale importance that standardized screening programs possess even further [45].

## 5. Conclusions

Our study analyzed a large cohort of patients and offers valuable real-world data, which show that multiple factors were positively associated with an increased risk of BC.

This study’s primary contribution lies in its ability to provide valuable real-world evidence from a previously unstudied population in Romania, bridging gaps in global breast cancer screening data. The comprehensive analysis of demographic, lifestyle, and clinical predictors, combined with an evaluation of regional disparities, has the potential to influence national screening protocols and offer insights for resource-limited countries or populations facing similar challenges. While certain unaccounted confounding variables may limit generalizability, the large cohort size and robust statistical approach provide a strong foundation for advancing both national and international breast cancer screening initiatives.

An older age, older age at first birth, and older menopausal age are all estrogen-dependent risk factors that were linked with an increased breast cancer risk in our study.

Furthermore, our findings concerning the rural/urban disparities and regional differences highlight the need for region-specific interventions to address lifestyle risk factors, improve healthcare access, and enhance breast cancer screening and follow-up protocols, particularly in underserved areas like the NE and SE regions.

## Figures and Tables

**Table 1:** Summary of numerical variables.

Variable	Median (Q25–Q75)
Age (years)	56 (53–62)
BMI (kg/m^2^)	28.12 (25.00–31.60)
Age at menarche (years)	14 (13–14)
Age at 1st birth (years)	22 (20–25)
Age at menopause (years)	49 (46–51)

Abbreviations: BMI—body mass index; Q25–Q75—interquartile range.

**Table 2:** Summary of categorical variables.

Variable	Class	Counts (Proportion)
FMH cancer	Yes	3638 (6.19%)
Hormonal treatment during menopause	Yes	3548 (6.04%)
Alcohol consumption	Yes	3032 (5.16%)
Smoking	Yes	9431 (16.05%)
Breastfeeding	Yes	55,024 (93.64%)
Breastfeeding period	<1 month	2450 (4.17%)
	<6 months	26,176 (44.55%)
	6–12 months	17,711 (30.14%)
	>12 months	12,423 (21.14%)
Vulnerability	Yes	25,672 (43.69%)
No. births	0	4076 (6.94%)
	1	18,468 (31.43%)
	2	26,218 (44.62%)
	3+	9998 (17.01%)
DPA	Yes	19,016 (32.36%)

Abbreviations: FMH—family medical history; DPA—daily physical activity.

**Table 3:** Comparison of continuous variables between rural and urban populations.

Variable	Rural (n = 24,508)	Urban (n = 34,252)	*p*-Value
Age	56.00 (53.00–61.00)	57.00 (53.00–62.00)	<0.001
BMI	28.58 (25.51–32.03)	27.73 (24.77–31.25)	<0.001
Age at menopause	49.00 (46.00–50.00)	49.00 (46.00–51.00)	<0.001
Age at 1st birth	21.00 (20.00–24.00)	22.00 (21.00–26.00)	<0.001
Age at menarche	14.00 (13.00–15.00)	14.00 (13.00–14.00)	<0.001

Abbreviations: BMI—body mass index.

**Table 4:** Comparison of categorical variables between rural and urban populations.

Variable	Group	Rural (n = 24,508)	Urban (n = 34,252)	*p*-Value
Vulnerability	Yes	24,481 (99.89%)	1191 (3.48%)	<0.001
Breastfeeding	Yes	23,047 (94.04%)	31,977 (93.36%)	<0.001
No. births	0	1227 (5.01%)	2849 (8.32%)	<0.001
	1	5583 (22.78%)	12,885 (37.62%)	
	2	11,238 (45.85%)	14,980 (43.73%)	
	3+	6460 (26.36%)	3538 (10.33%)	
Breastfeeding period	<1 month	911 (3.72%)	1539 (4.49%)	<0.001
	1–6 months	9493 (38.73%)	16,683 (48.71%)	
	6–12 months	7327 (29.9%)	10,384 (30.32%)	
	>12 months	6777 (27.65%)	5646 (16.48%)	
Hormonal treatment during menopause	Yes	1304 (5.32%)	2244 (6.55%)	<0.001
DPA	Yes	8545 (34.87%)	10,471 (30.57%)	<0.001
Alcohol consumption	Yes	1355 (5.53%)	1677 (4.9%)	<0.001
Smoking	Yes	3256 (13.29%)	6175 (18.03%)	<0.001
FMH cancer	Yes	1309 (5.34%)	2329 (6.8%)	<0.001

Abbreviations: FMH—family medical history; DPA—daily physical activity.

**Table 5:** Mammography results by rural and urban populations.

Variable	Group	Rural (n = 24,508)	Urban (n = 34,252)	*p*-Value
Mammography	Positive	1347 (5.5%)	2430 (7.09%)	<0.001

**Table 6:** Echography results by rural and urban populations.

Variable	Group	Rural (n = 1062)	Urban (n = 2135)	*p*-Value
Echography	Positive	314 (29.56%)	596 (27.91%)	<0.001

**Table 7:** Biopsy results by rural and urban populations.

Variable	Group	Rural (n = 253)	Urban (n = 435)	*p*-Value
Biopsy	Positive	162 (64.03%)	308 (70.81%)	0.07

**Table 8:** Comparison of continuous variables across regions.

Variable	NE (n = 15,219)	NW (n = 27,136)	SE (n = 8668)	W (n = 7737)	*p*-Value
Age	56.00 (53.00–62.00)	57.00 (53.00–62.00)	56.00 (53.00–62.00)	57.00 (53.00–62.00)	<0.001
BMI	28.34 (25.34–31.64)	28.12 (25.00–31.64)	27.68 (24.78–31.24)	27.92 (24.80–31.49)	<0.001
Age at menopause	49.00 (46.00–50.00)	49.00 (46.00–51.00)	49.00 (46.00–50.00)	49.00 (46.00–51.00)	<0.001
Age at 1st birth	22.00 (20.00–25.00)	22.00 (20.00–25.00)	22.00 (20.00–24.00)	22.00 (21.00–26.00)	<0.001
Age at menarche	14.00 (13.00–14.00)	14.00 (13.00–14.00)	14.00 (13.00–14.00)	14.00 (13.00–14.00)	<0.001

Abbreviations: BMI—body mass index.

**Table 9:** Comparison of categorical variables across regions.

Variable	Group	NE (n = 15,219)	NW (n = 27,136)	SE (n = 8668)	W (n = 7737)	*p*-Value
Vulnerability	Yes	7726 (50.77%)	10,153 (37.42%)	5485 (63.28%)	2308 (29.83%)	<0.001
Breastfeeding	Yes	14,332 (94.17%)	25,434 (93.73%)	8064 (93.03%)	7194 (92.98%)	<0.001
No. births	0	1032 (6.78%)	1773 (6.53%)	537 (6.2%)	734 (9.49%)	<0.001
	1	4054 (26.64%)	8484 (31.26%)	2558 (29.51%)	3372 (43.58%)	
	2	6658 (43.75%)	12,979 (47.83%)	3718 (42.89%)	2863 (37%)	
	3+	3475 (22.84%)	3900 (14.37%)	1855 (52.13%)	768 (9.92%)	
Breastfeeding period	<1 month	436 (2.86%)	1224 (4.51%)	243 (2.8%)	547 (7.07%)	<0.001
	1–6 months	5892 (38.71%)	13,098 (48.27%)	2922 (33.71%)	4264 (55.11%)	
	6–12 months	4923 (32.35%)	7967 (29.36%)	3001 (34.62%)	1820 (23.52%)	
	>12 months	3968 (26.07%)	4847 (17.86%)	2502 (28.86%)	1106 (14.29%)	
Hormonal treatment during menopause	Yes	879 (5.78%)	1889 (6.96%)	312 (3.6%)	468 (6.05%)	<0.001
DPA	Yes	4377 (28.76%)	8161 (30.07%)	2469 (28.48%)	4009 (51.82%)	<0.001
Alcohol consumption	Yes	1926 (12.66%)	322 (1.19%)	694 (8.01%)	90 (1.16%)	<0.001
Smoking	Yes	1844 (12.12%)	4937 (18.19%)	1142 (13.17%)	1508 (19.49%)	<0.001
FMH cancer	Yes	1068 (7.02%)	1572 (5.79%)	440 (5.08%)	558 (7.21%)	<0.001

Abbreviations: FMH—family medical history; DPA—daily physical activity.

**Table 10:** Mammography results by region.

Variable	Group	NE (n = 15,219)	NW (n = 27,136)	SE (n = 8668)	W (n = 7737)	*p*-Value
Mammography	Positive	1013 (6.66%)	1511 (5.57%)	473 (5.46%)	780 (10.08%)	<0.001

**Table 11:** Echography results by region.

Variable	Group	NE (n = 886)	NW (n = 1318)	SE (n = 298)	W (n = 695)	*p*-Value
Echography	Positive	247 (27.87%)	408 (30.96%)	42 (14.09%)	213 (30.65%)	<0.001

**Table 12:** Biopsy results by region.

Variable	Group	NE (n = 207)	NW (n = 341)	SE (n = 33)	W (n = 107)	*p*-Value
Biopsy	Positive	149 (71.98%)	229 (67.16%)	18 (54.55%)	74 (69.16%)	0.22

**Table 13:** Comparison of continuous variables by mammography positivity.

Variable	Mammography Positive (n = 3777)	Mammography Negative (n = 54,983)	*p*-Value
Age	56.00 (53.00–62.00)	56.00 (53.00–62.00)	0.02
BMI	27.43 (24.38–31.14)	28.12 (25.07–31.63)	<0.001
Age at menopause	49.00 (47.00–51.00)	49.00 (46.00–51.00)	<0.001
Age at 1st birth	22.00 (21.00–25.00)	22.00 (20.00–25.00)	<0.001
Age at menarche	14.00 (13.00–14.00)	14.00 (13.00–14.00)	0.16

Abbreviations: BMI—body mass index.

**Table 14:** Comparison of categorical variables by mammography positivity.

Variable	Group	Mammography Positive (n = 3777)	Mammography Negative (n = 54,983)	*p*-Value
Vulnerability	Yes	1422 (37.65%)	24,250 (44.1%)	<0.001
Breastfeeding	Yes	3501 (92.69%)	51,523 (93.71%)	0.01
No. births	0	368 (9.74%)	3708 (6.74%)	<0.001
	1	1308 (34.63%)	17,160 (31.21%)	
	2	1613 (42.71%)	24,605 (44.75%)	
	3+	488 (12.92%)	9510 (17.3%)	
Breastfeeding period	<1 month	169 (4.47%)	2281 (4.15%)	<0.001
	1–6 months	1812 (47.97%)	24,364 (44.31%)	
	6–12 months	1101 (29.15%)	16,610 (30.21%)	
	>12 months	695 (18.4%)	11,728 (21.33%)	
Hormonal treatment during menopause	Yes	208 (5.51%)	3340 (6.07%)	0.16
DPA	Yes	1221 (32.33%)	17,795 (32.36%)	0.96
Alcohol consumption	Yes	212 (5.61%)	2820 (5.13%)	0.19
Smoking	Yes	685 (18.14%)	8746 (15.91%)	<0.001
FMH cancer	Yes	291 (7.7%)	3347 (6.09%)	<0.001

Abbreviations: FMH—family medical history; DPA—daily physical activity.

**Table 15:** Comparison of continuous variables by age groups.

**Variable**	**<50 Years** **(n = 3893)**	**50–60 Years** **(n = 36,724)**	**>60 Years** **(n = 18,143)**	***p*-Value**
BMI	26.99 (24.02–30.42)	27.92 (24.88–31.53)	28.67 (25.64–32.02)	<0.001
Age at menopause	49.00 (47.00–49.00)	49.00 (46.00–51.00)	49.00 (45.00–52.00)	<0.001
Age at 1st birth	23.00 (21.00–27.00)	22.00 (20.00–25.00)	22.00 (20.00–24.00)	<0.001
Age at menarche	14.00 (13.00–14.00)	14.00 (13.00–14.00)	14.00 (13.00–14.00)	<0.001

Abbreviations: BMI—body mass index.

**Table 16:** Comparison of categorical variables by age groups.

Variable	Group	<50 Years (n = 3893)	50–60 Years (n = 36,724)	>60 Years (n = 18,143)	*p*-Value
Vulnerability	Yes	1896 (48.7%)	16,541 (45.04%)	7235 (39.88%)	<0.001
Breastfeeding	Yes	3608 (92.68%)	34,358 (93.56%)	17,058 (94.02%)	0.004
No. births	0	328 (8.43%)	2673 (7.28%)	1075 (5.93%)	<0.001
	1	1433 (36.81%)	12,717 (34.63%)	4318 (23.8%)	
	2	1679 (43.13%)	16,065 (43.75%)	8474 (46.71%)	
	3+	453 (11.64%)	5269 (14.34%)	4276 (23.57%)	
Breastfeeding period	<1 month	172 (4.42%)	1647 (4.48%)	631 (3.48%)	<0.001
	1–6 months	1683 (43.23%)	16,389 (44.63%)	8104 (44.67%)	
	6–12 months	1103 (28.33%)	10,910 (29.71%)	5698 (31.41%)	
	>12 months	935 (24.02%)	7778 (21.18%)	3710 (20.45%)	
Hormonal treatment during menopause	Yes	190 (4.88%)	2268 (6.18%)	1090 (6.01%)	0.005
DPA	Yes	1307 (33.57%)	11,781 (32.08%)	5928 (32.67%)	0.09
Alcohol consumption	Yes	219 (5.63%)	1918 (5.22%)	895 (4.93%)	0.14
Smoking	Yes	744 (19.11%)	6487 (17.66%)	2200 (12.13%)	<0.001
FMH cancer	Yes	236 (6.06%)	2162 (5.89%)	1240 (6.83%)	<0.001

Abbreviations: FMH—family medical history; DPA—daily physical activity.

**Table 17:** Mammography results by age groups.

Variable	Group	<50 Years (n = 3893)	50–60 Years (n = 36,724)	>60 Years (n = 18,143)	*p*-Value
Mammography	Positive	283 (7.27%)	2331 (6.35%)	1163 (6.41%)	<0.001

**Table 18:** Echography results by age groups.

Variable	Group	<50 Years (n = 250)	50–60 Years (n = 1970)	>60 Years (n = 977)	*p*-Value
Echography	Positive	55 (22%)	459 (23.3%)	396 (40.53%)	<0.001

**Table 19:** Biopsy results by age groups.

Variable	Group	<50 Years (n = 39)	50–60 Years (n = 342)	>60 Years (n = 307)	*p*-Value
Biopsy	Positive	18 (46.15%)	219 (64.04%)	233 (75.9%)	<0.001

**Table 20:** Comparison of continuous variables by BMI groups.

Variable	Underweight (n = 338)	Normal Weight (n = 14,452)	Overweight (n = 22,865)	Obese (n = 21,105)	*p*-Value
Age	55.00 (52.00–60.00)	55.00 (52.00–61.00)	56.00 (53.00–62.00)	57.00 (53.00–62.00)	<0.001
Age at menopause	48.50 (45.00–50.00)	49.00 (46.00–50.00)	49.00 (46.00–51.00)	49.00 (46.00–51.00)	<0.001
Age at 1st birth	22.00 (20.00–26.00)	22.00 (21.00–26.00)	22.00 (20.00–25.00)	22.00 (20.00–24.00)	<0.001
Age at menarche	14.00 (13.00–15.00)	14.00 (13.00–14.00)	14.00 (13.00–14.00)	14.00 (13.00–14.00)	<0.001

**Table 21:** Comparison of categorical variables by BMI groups.

Variable	Group	Underweight (n = 338)	Normal Weight (n = 14,452)	Overweight (n = 22,865)	Obese (n = 21,105)	*p*-Value
Vulnerability	Yes	152 (44.97%)	5471 (37.86%)	9915 (43.36%)	10,134 (48.02%)	<0.001
Breastfeeding	Yes	313 (92.6%)	13,549 (93.75%)	21,501 (94.03%)	19,661 (93.16%)	0.001
No. births	0	38 (11.24%)	1320 (9.13%)	1484 (6.49%)	1234 (5.85%)	<0.001
	1	125 (36.98%)	5398 (37.35%)	7235 (31.64%)	5710 (27.06%)	
	2	122 (36.09%)	5902 (40.84%)	10,429 (45.61%)	9765 (46.27%)	
	3+	53 (15.68%)	1832 (12.68%)	3717 (16.26%)	4396 (20.83%)	
Breastfeeding period	<1 month	15 (4.44%)	566 (3.92%)	961 (4.2%)	908 (4.3%)	<0.001
	1–6 months	185 (54.73%)	6818 (47.18%)	10,062 (44.01%)	9111 (43.17%)	
	6–12 months	72 (21.3%)	4391 (30.38%)	7017 (30.69%)	6231 (29.52%)	
	>12 months	66 (19.53%)	2677 (18.52%)	4825 (21.1%)	4855 (23%)	
Hormonal treatment during menopause	Yes	16 (4.73%)	929 (6.43%)	1385 (6.06%)	1218 (5.77%)	0.06
DPA	Yes	135 (39.94%)	5610 (38.82%)	7579 (33.15%)	5692 (26.97%)	<0.001
Alcohol consumption	Yes	15 (4.44%)	762 (5.27%)	1213 (5.31%)	1042 (4.94%)	0.28
Smoking	Yes	119 (35.21%)	3123 (21.61%)	3464 (15.15%)	2725 (12.91%)	<0.001
FMH cancer	Yes	20 (5.92%)	910 (6.3%)	1354 (5.92%)	1354 (6.42%)	0.17

Abbreviations: FMH—family medical history; DPA—daily physical activity.

**Table 22:** Mammography results by BMI groups.

Variable	Group	Underweight (n = 338)	Normal Weight (n = 14,452)	Overweight (n = 22,865)	Obese (n = 21,105)	*p*-Value
Mammography	Positive	29 (8.58%)	1121 (7.76%)	1440 (6.3%)	1187 (5.62%)	<0.001

**Table 23:** Echography results by BMI groups.

Variable	Group	Underweight (n = 19)	Normal Weight (n = 942)	Overweight (n = 1211)	Obese (n = 1025)	*p*-Value
Echography	Positive	6 (31.58%)	188 (19.96%)	338 (27.91%)	378 (36.88%)	<0.001

**Table 24:** Biopsy results by BMI groups.

Variable	Group	Underweight (n = 4)	Normal Weight (n = 132)	Overweight (n = 248)	Obese (n = 304)	*p*-Value
Biopsy	Positive	4 (100%)	90 (68.18%)	159 (64.11%)	217 (71.38%)	0.16

**Table 25:** Logistic regression analysis of predictors for progression from mammography to echography.

Predictors	OR	CI	*p*-Value
BMI	0.98	0.98–0.99	<0.001
FMH cancer [Yes]	1.28	1.11–1.46	<0.001
Age at 1st birth	1.03	1.02–1.03	<0.001
Age at menopause	1.02	1.01–1.02	<0.001
R^2^ Nagelkerke = 0.004			

Abbreviations: FMH—family medical history, OR—odds ratio, CI—95% confidence interval.

**Table 26:** Logistic regression analysis of predictors for progression from echography to biopsy.

Predictors	OR	CI	*p*-Value
Age	1.08	1.05–1.11	<0.001
Age 1st birth	1.04	1.00–1.08	<0.001
BMI	1.02	0.99–1.05	0.286
R^2^ Nagelkerke = 0.06			

Abbreviations: BMI—body mass index, OR—odds ratio, CI—95% confidence interval.

## Data Availability

Further information concerning the present article is available from the corresponding author upon reasonable request.
